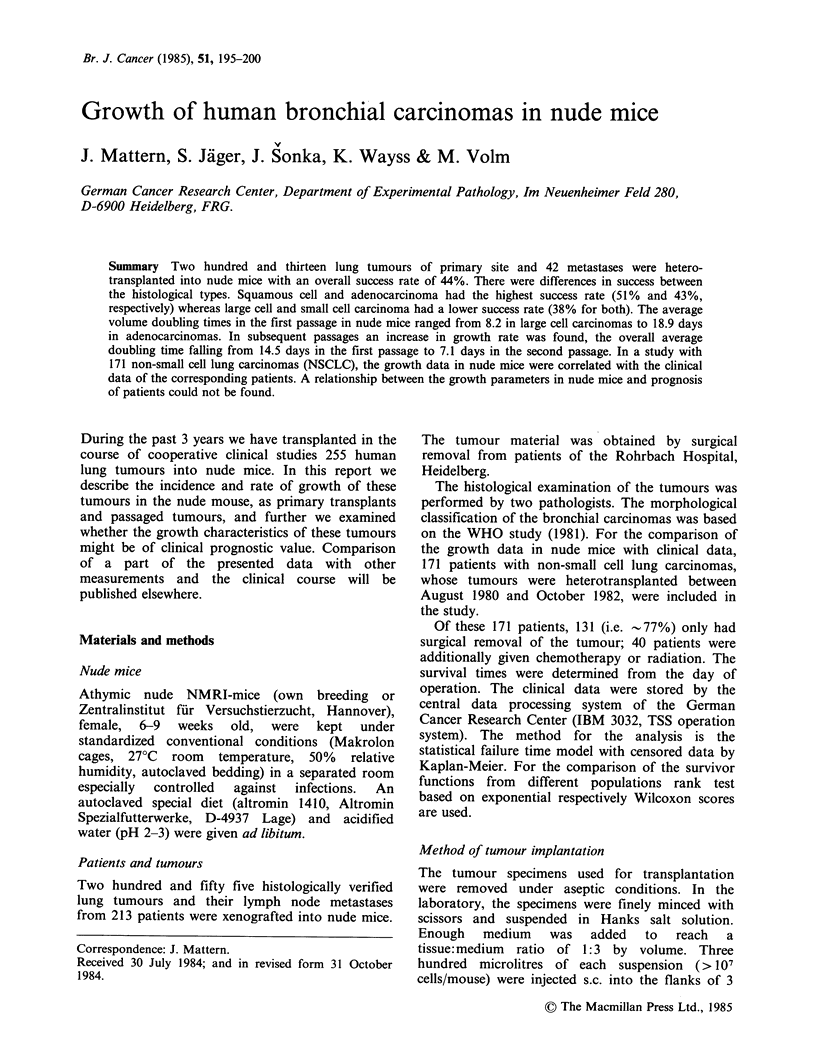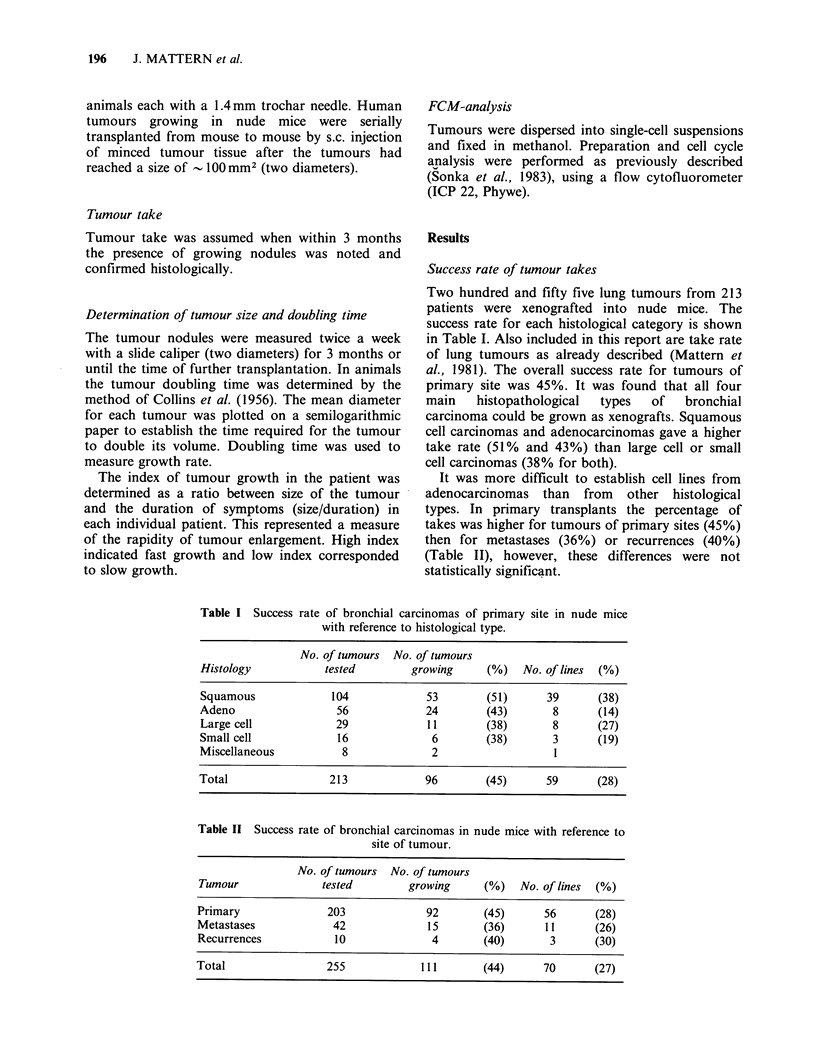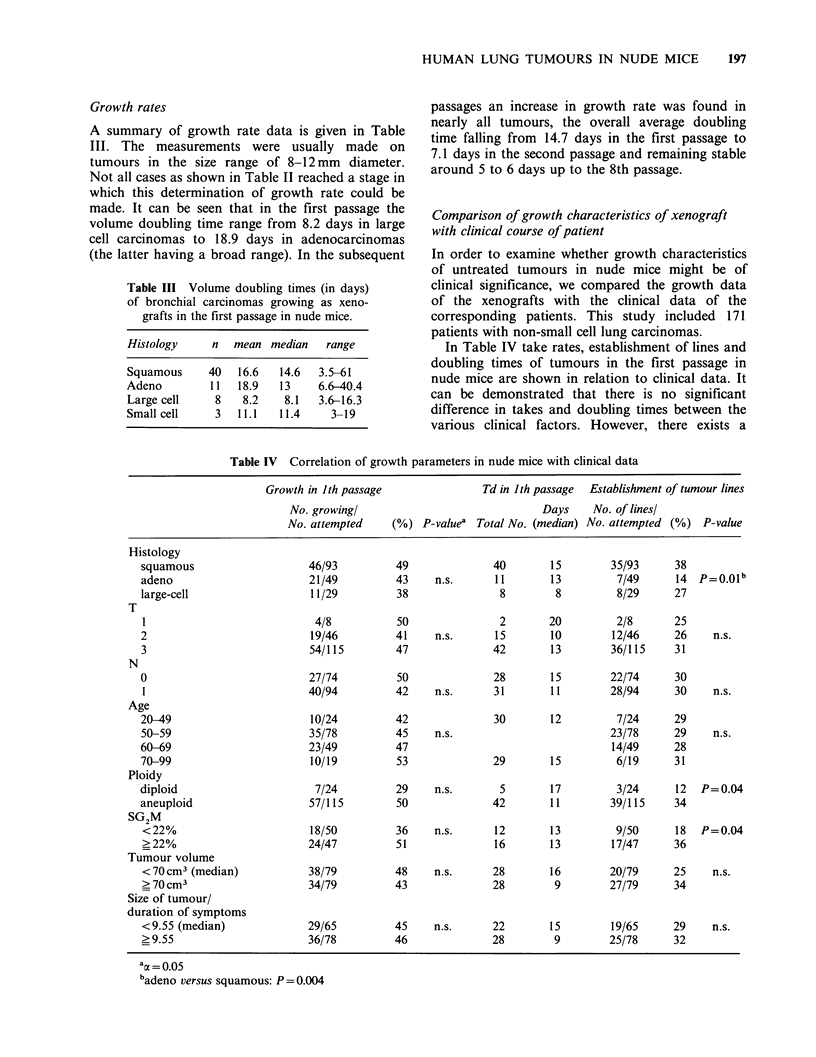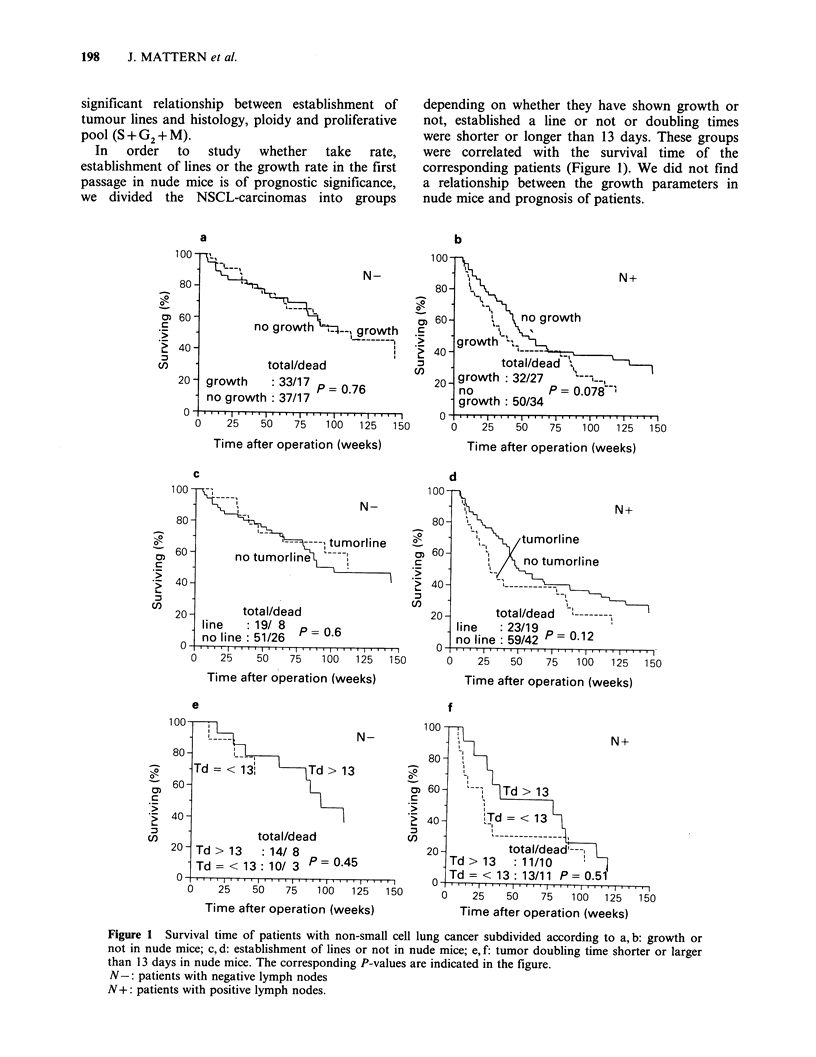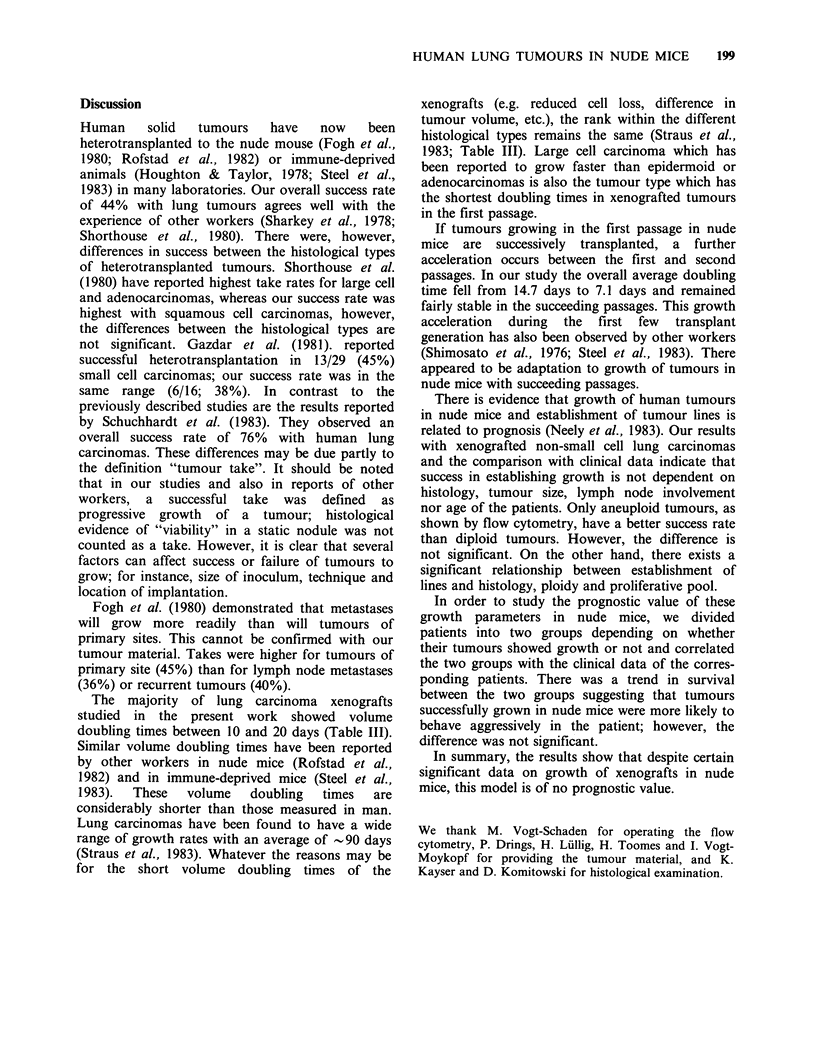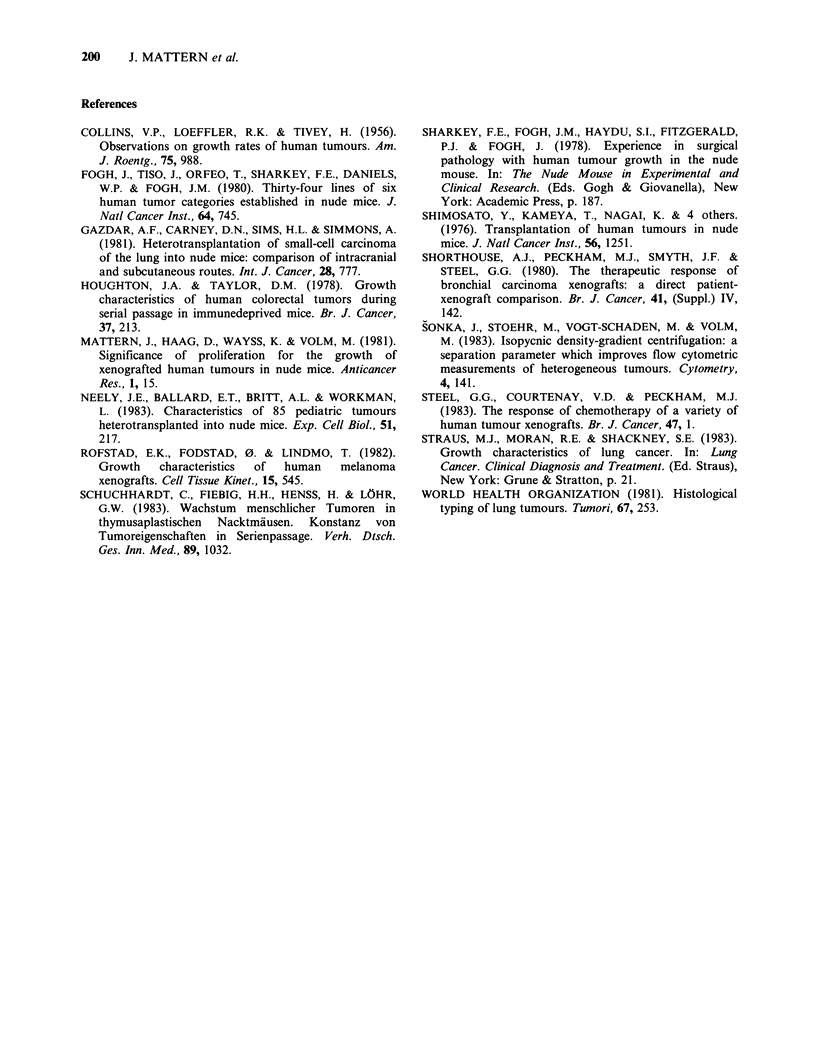# Growth of human bronchial carcinomas in nude mice.

**DOI:** 10.1038/bjc.1985.29

**Published:** 1985-02

**Authors:** J. Mattern, S. Jäger, J. Sonka, K. Wayss, M. Volm

## Abstract

Two hundred and thirteen lung tumours of primary site and 42 metastases were heterotransplanted into nude mice with an overall success rate of 44%. There were differences in success between the histological types. Squamous cell and adenocarcinoma had the highest success rate (51% and 43%, respectively) whereas large cell and small cell carcinoma had a lower success rate (38% for both). The average volume doubling times in the first passage in nude mice ranged from 8.2 in large cell carcinomas to 18.9 days in adenocarcinomas. In subsequent passages an increase in growth rate was found, the overall average doubling time falling from 14.5 days in the first passage to 7.1 days in the second passage. In a study with 171 non-small cell lung carcinomas (NSCLC), the growth data in nude mice were correlated with the clinical data of the corresponding patients. A relationship between the growth parameters in nude mice and prognosis of patients could not be found.


*Br. J. Cancer* (1985), **51**, 195–200

### Growth of human bronchial carcinomas in nude mice

J. Mattern, S. Jäger, J. Šonka, K. Wayss & M. Volm

*German Cancer Research Center, Department of Experimental Pathology, Im Neuenheimer Feld 280, D-6900 Heidelberg, FRG.*

**Summary** Two hundred and thirteen lung tumours of primary site and 42 metastases were heterotransplanted into nude mice with an overall success rate of 44%. There were differences in success between the histological types. Squamous cell and adenocarcinoma had the highest success rate (51% and 43%, respectively) whereas large cell and small cell carcinoma had a lower success rate (38% for both). The average volume doubling times in the first passage in nude mice ranged from 8.2 in large cell carcinomas to 18.9 days in adenocarcinomas. In subsequent passages an increase in growth rate was found, the overall average doubling time falling from 14.5 days in the first passage to 7.1 days in the second passage. In a study with 171 non-small cell lung carcinomas (NSCLC), the growth data in nude mice were correlated with the clinical data of the corresponding patients. A relationship between the growth parameters in nude mice and prognosis of patients could not be found.

During the past 3 years we have transplanted in the course of cooperative clinical studies 255 human lung tumours into nude mice. In this report we describe the incidence and rate of growth of these tumours in the nude mouse, as primary transplants and passaged tumours, and further we examined whether the growth characteristics of these tumours might be of clinical prognostic value. Comparison of a part of the presented data with other measurements and the clinical course will be published elsewhere.

### Materials and methods

### *Nude mice*

Athymic nude NMRI-mice (own breeding or Zentralinstitut für Versuchstierzucht, Hannover), female, 6–9 weeks old, were kept under standardized conventional conditions (Makrolon cages, 27°C room temperature, 50% relative humidity, autoclaved bedding) in a separated room especially controlled against infections. An autoclaved special diet (altromin 1410, Altromin Spezialfutterwerke, D-4937 Lage) and acidified water (pH 2–3) were given *ad libitum*.

### *Patients and tumours*

Two hundred and fifty five histologically verified lung tumours and their lymph node metastases from 213 patients were xenografted into nude mice. The tumour material was obtained by surgical removal from patients of the Rohrbach Hospital, Heidelberg.

The histological examination of the tumours was performed by two pathologists. The morphological classification of the bronchial carcinomas was based on the WHO study (1981). For the comparison of the growth data in nude mice with clinical data, 171 patients with non-small cell lung carcinomas, whose tumours were heterotransplanted between August 1980 and October 1982, were included in the study.

Of these 171 patients, 131 (i.e. ~77%) only had surgical removal of the tumour; 40 patients were additionally given chemotherapy or radiation. The survival times were determined from the day of operation. The clinical data were stored by the central data processing system of the German Cancer Research Center (IBM 3032, TSS operation system). The method for the analysis is the statistical failure time model with censored data by Kaplan-Meier. For the comparison of the survivor functions from different populations rank test based on exponential respectively Wilcoxon scores are used.

### *Method of tumour implantation*

The tumour specimens used for transplantation were removed under aseptic conditions. In the laboratory, the specimens were finely minced with scissors and suspended in Hanks salt solution. Enough medium was added to reach a tissue:medium ratio of 1:3 by volume. Three hundred microlitres of each suspension ($>10^7$ cells/mouse) were injected s.c. into the flanks of 3

Correspondence: J. Mattern.
Received 30 July 1984; and in revised form 31 October 1984.

© The Macmillan Press Ltd., 1985

animals each with a 1.4 mm trochar needle. Human tumours growing in nude mice were serially transplanted from mouse to mouse by s.c. injection of minced tumour tissue after the tumours had reached a size of ~100 mm² (two diameters).

### Tumour take

Tumour take was assumed when within 3 months the presence of growing nodules was noted and confirmed histologically.

### Determination of tumour size and doubling time

The tumour nodules were measured twice a week with a slide caliper (two diameters) for 3 months or until the time of further transplantation. In animals the tumour doubling time was determined by the method of Collins *et al.* (1956). The mean diameter for each tumour was plotted on a semilogarithmic paper to establish the time required for the tumour to double its volume. Doubling time was used to measure growth rate.

The index of tumour growth in the patient was determined as a ratio between size of the tumour and the duration of symptoms (size/duration) in each individual patient. This represented a measure of the rapidity of tumour enlargement. High index indicated fast growth and low index corresponded to slow growth.

### FCM-analysis

Tumours were dispersed into single-cell suspensions and fixed in methanol. Preparation and cell cycle analysis were performed as previously described (Šonka *et al.*, 1983), using a flow cytofluorometer (ICP 22, Phywe).

### Results

### Success rate of tumour takes

Two hundred and fifty five lung tumours from 213 patients were xenografted into nude mice. The success rate for each histological category is shown in Table I. Also included in this report are take rate of lung tumours as already described (Mattern *et al.*, 1981). The overall success rate for tumours of primary site was 45%. It was found that all four main histopathological types of bronchial carcinoma could be grown as xenografts. Squamous cell carcinomas and adenocarcinomas gave a higher take rate (51% and 43%) than large cell or small cell carcinomas (38% for both).

It was more difficult to establish cell lines from adenocarcinomas than from other histological types. In primary transplants the percentage of takes was higher for tumours of primary sites (45%) then for metastases (36%) or recurrences (40%) (Table II), however, these differences were not statistically significant.

**Table I** Success rate of bronchial carcinomas of primary site in nude mice with reference to histological type.

| *Histology* | *No. of tumours tested* | *No. of tumours growing* | (%) | *No. of lines* | (%) |
|---|---|---|---|---|---|
| Squamous | 104 | 53 | (51) | 39 | (38) |
| Adeno | 56 | 24 | (43) | 8 | (14) |
| Large cell | 29 | 11 | (38) | 8 | (27) |
| Small cell | 16 | 6 | (38) | 3 | (19) |
| Miscellaneous | 8 | 2 | | 1 | |
| Total | 213 | 96 | (45) | 59 | (28) |

**Table II** Success rate of bronchial carcinomas in nude mice with reference to site of tumour.

| *Tumour* | *No. of tumours tested* | *No. of tumours growing* | (%) | *No. of lines* | (%) |
|---|---|---|---|---|---|
| Primary | 203 | 92 | (45) | 56 | (28) |
| Metastases | 42 | 15 | (36) | 11 | (26) |
| Recurrences | 10 | 4 | (40) | 3 | (30) |
| Total | 255 | 111 | (44) | 70 | (27) |

*Growth rates*

A summary of growth rate data is given in Table III. The measurements were usually made on tumours in the size range of 8–12 mm diameter. Not all cases as shown in Table II reached a stage in which this determination of growth rate could be made. It can be seen that in the first passage the volume doubling time range from 8.2 days in large cell carcinomas to 18.9 days in adenocarcinomas (the latter having a broad range). In the subsequent passages an increase in growth rate was found in nearly all tumours, the overall average doubling time falling from 14.7 days in the first passage to 7.1 days in the second passage and remaining stable around 5 to 6 days up to the 8th passage.

**Table III** Volume doubling times (in days) of bronchial carcinomas growing as xenografts in the first passage in nude mice.

| *Histology* | *n* | *mean* | *median* | *range* |
|---|---|---|---|---|
| Squamous | 40 | 16.6 | 14.6 | 3.5–61 |
| Adeno | 11 | 18.9 | 13 | 6.6–40.4 |
| Large cell | 8 | 8.2 | 8.1 | 3.6–16.3 |
| Small cell | 3 | 11.1 | 11.4 | 3–19 |

*Comparison of growth characteristics of xenograft with clinical course of patient*

In order to examine whether growth characteristics of untreated tumours in nude mice might be of clinical significance, we compared the growth data of the xenografts with the clinical data of the corresponding patients. This study included 171 patients with non-small cell lung carcinomas.

In Table IV take rates, establishment of lines and doubling times of tumours in the first passage in nude mice are shown in relation to clinical data. It can be demonstrated that there is no significant difference in takes and doubling times between the various clinical factors. However, there exists a

**Table IV** Correlation of growth parameters in nude mice with clinical data

| | *Growth in 1th passage* | | | *Td in 1th passage* | | *Establishment of tumour lines* | | |
|---|---|---|---|---|---|---|---|---|
| | *No. growing/ No. attempted* | (%) | *P-value*[a] | *Total No.* | *Days (median)* | *No. of lines/ No. attempted* | (%) | *P-value* |
| Histology | | | | | | | | |
| squamous | 46/93 | 49 | | 40 | 15 | 35/93 | 38 | |
| adeno | 21/49 | 43 | n.s. | 11 | 13 | 7/49 | 14 | $P=0.01$[b] |
| large-cell | 11/29 | 38 | | 8 | 8 | 8/29 | 27 | |
| T | | | | | | | | |
| 1 | 4/8 | 50 | | 2 | 20 | 2/8 | 25 | |
| 2 | 19/46 | 41 | n.s. | 15 | 10 | 12/46 | 26 | n.s. |
| 3 | 54/115 | 47 | | 42 | 13 | 36/115 | 31 | |
| N | | | | | | | | |
| 0 | 27/74 | 50 | | 28 | 15 | 22/74 | 30 | |
| 1 | 40/94 | 42 | n.s. | 31 | 11 | 28/94 | 30 | n.s. |
| Age | | | | | | | | |
| 20–49 | 10/24 | 42 | | 30 | 12 | 7/24 | 29 | |
| 50–59 | 35/78 | 45 | n.s. | | | 23/78 | 29 | n.s. |
| 60–69 | 23/49 | 47 | | | | 14/49 | 28 | |
| 70–99 | 10/19 | 53 | | 29 | 15 | 6/19 | 31 | |
| Ploidy | | | | | | | | |
| diploid | 7/24 | 29 | n.s. | 5 | 17 | 3/24 | 12 | $P=0.04$ |
| aneuploid | 57/115 | 50 | | 42 | 11 | 39/115 | 34 | |
| $SG_2M$ | | | | | | | | |
| <22% | 18/50 | 36 | n.s. | 12 | 13 | 9/50 | 18 | $P=0.04$ |
| ≧22% | 24/47 | 51 | | 16 | 13 | 17/47 | 36 | |
| Tumour volume | | | | | | | | |
| <70 $cm^3$ (median) | 38/79 | 48 | n.s. | 28 | 16 | 20/79 | 25 | n.s. |
| ≧70 $cm^3$ | 34/79 | 43 | | 28 | 9 | 27/79 | 34 | |
| Size of tumour/ duration of symptoms | | | | | | | | |
| <9.55 (median) | 29/65 | 45 | n.s. | 22 | 15 | 19/65 | 29 | n.s. |
| ≧9.55 | 36/78 | 46 | | 28 | 9 | 25/78 | 32 | |

[a] $\alpha=0.05$
[b] adeno *versus* squamous: $P=0.004$

significant relationship between establishment of tumour lines and histology, ploidy and proliferative pool ($S+G_2+M$).

In order to study whether take rate, establishment of lines or the growth rate in the first passage in nude mice is of prognostic significance, we divided the NSCL-carcinomas into groups depending on whether they have shown growth or not, established a line or not or doubling times were shorter or longer than 13 days. These groups were correlated with the survival time of the corresponding patients (Figure 1). We did not find a relationship between the growth parameters in nude mice and prognosis of patients.

**Figure 1** Survival time of patients with non-small cell lung cancer subdivided according to a, b: growth or not in nude mice; c, d: establishment of lines or not in nude mice; e, f: tumor doubling time shorter or larger than 13 days in nude mice. The corresponding *P*-values are indicated in the figure.
*N*−: patients with negative lymph nodes
*N*+: patients with positive lymph nodes.

### Discussion

Human solid tumours have now been heterotransplanted to the nude mouse (Fogh *et al.*, 1980; Rofstad *et al.*, 1982) or immune-deprived animals (Houghton & Taylor, 1978; Steel *et al.*, 1983) in many laboratories. Our overall success rate of 44% with lung tumours agrees well with the experience of other workers (Sharkey *et al.*, 1978; Shorthouse *et al.*, 1980). There were, however, differences in success between the histological types of heterotransplanted tumours. Shorthouse *et al.* (1980) have reported highest take rates for large cell and adenocarcinomas, whereas our success rate was highest with squamous cell carcinomas, however, the differences between the histological types are not significant. Gazdar *et al.* (1981). reported successful heterotransplantation in 13/29 (45%) small cell carcinomas; our success rate was in the same range (6/16; 38%). In contrast to the previously described studies are the results reported by Schuchhardt *et al.* (1983). They observed an overall success rate of 76% with human lung carcinomas. These differences may be due partly to the definition "tumour take". It should be noted that in our studies and also in reports of other workers, a successful take was defined as progressive growth of a tumour; histological evidence of "viability" in a static nodule was not counted as a take. However, it is clear that several factors can affect success or failure of tumours to grow; for instance, size of inoculum, technique and location of implantation.

Fogh *et al.* (1980) demonstrated that metastases will grow more readily than will tumours of primary sites. This cannot be confirmed with our tumour material. Takes were higher for tumours of primary site (45%) than for lymph node metastases (36%) or recurrent tumours (40%).

The majority of lung carcinoma xenografts studied in the present work showed volume doubling times between 10 and 20 days (Table III). Similar volume doubling times have been reported by other workers in nude mice (Rofstad *et al.*, 1982) and in immune-deprived mice (Steel *et al.*, 1983). These volume doubling times are considerably shorter than those measured in man. Lung carcinomas have been found to have a wide range of growth rates with an average of $\sim$90 days (Straus *et al.*, 1983). Whatever the reasons may be for the short volume doubling times of the xenografts (e.g. reduced cell loss, difference in tumour volume, etc.), the rank within the different histological types remains the same (Straus *et al.*, 1983; Table III). Large cell carcinoma which has been reported to grow faster than epidermoid or adenocarcinomas is also the tumour type which has the shortest doubling times in xenografted tumours in the first passage.

If tumours growing in the first passage in nude mice are successively transplanted, a further acceleration occurs between the first and second passages. In our study the overall average doubling time fell from 14.7 days to 7.1 days and remained fairly stable in the succeeding passages. This growth acceleration during the first few transplant generation has also been observed by other workers (Shimosato *et al.*, 1976; Steel *et al.*, 1983). There appeared to be adaptation to growth of tumours in nude mice with succeeding passages.

There is evidence that growth of human tumours in nude mice and establishment of tumour lines is related to prognosis (Neely *et al.*, 1983). Our results with xenografted non-small cell lung carcinomas and the comparison with clinical data indicate that success in establishing growth is not dependent on histology, tumour size, lymph node involvement nor age of the patients. Only aneuploid tumours, as shown by flow cytometry, have a better success rate than diploid tumours. However, the difference is not significant. On the other hand, there exists a significant relationship between establishment of lines and histology, ploidy and proliferative pool.

In order to study the prognostic value of these growth parameters in nude mice, we divided patients into two groups depending on whether their tumours showed growth or not and correlated the two groups with the clinical data of the corresponding patients. There was a trend in survival between the two groups suggesting that tumours successfully grown in nude mice were more likely to behave aggressively in the patient; however, the difference was not significant.

In summary, the results show that despite certain significant data on growth of xenografts in nude mice, this model is of no prognostic value.

We thank M. Vogt-Schaden for operating the flow cytometry, P. Drings, H. Lüllig, H. Toomes and I. Vogt-Moykopf for providing the tumour material, and K. Kayser and D. Komitowski for histological examination.

### References

COLLINS, V.P., LOEFFLER, R.K. & TIVEY, H. (1956). Observations on growth rates of human tumours. *Am. J. Roentg.*, **75**, 988.

FOGH, J., TISO, J., ORFEO, T., SHARKEY, F.E., DANIELS, W.P. & FOGH, J.M. (1980). Thirty-four lines of six human tumor categories established in nude mice. *J. Natl Cancer Inst.*, **64**, 745.

GAZDAR, A.F., CARNEY, D.N., SIMS, H.L. & SIMMONS, A. (1981). Heterotransplantation of small-cell carcinoma of the lung into nude mice: comparison of intracranial and subcutaneous routes. *Int. J. Cancer*, **28**, 777.

HOUGHTON, J.A. & TAYLOR, D.M. (1978). Growth characteristics of human colorectal tumors during serial passage in immunedeprived mice. *Br. J. Cancer*, **37**, 213.

MATTERN, J., HAAG, D., WAYSS, K. & VOLM, M. (1981). Significance of proliferation for the growth of xenografted human tumours in nude mice. *Anticancer Res.*, **1**, 15.

NEELY, J.E., BALLARD, E.T., BRITT, A.L. & WORKMAN, L. (1983). Characteristics of 85 pediatric tumours heterotransplanted into nude mice. *Exp. Cell Biol.*, **51**, 217.

ROFSTAD, E.K., FODSTAD, Ø. & LINDMO, T. (1982). Growth characteristics of human melanoma xenografts. *Cell Tissue Kinet.*, **15**, 545.

SCHUCHHARDT, C., FIEBIG, H.H., HENSS, H. & LÖHR, G.W. (1983). Wachstum menschlicher Tumoren in thymusaplastischen Nacktmäusen. Konstanz von Tumoreigenschaften in Serienpassage. *Verh. Dtsch. Ges. Inn. Med.*, **89**, 1032.

SHARKEY, F.E., FOGH, J.M., HAYDU, S.I., FITZGERALD, P.J. & FOGH, J. (1978). Experience in surgical pathology with human tumour growth in the nude mouse. In: *The Nude Mouse in Experimental and Clinical Research*. (Eds. Gogh & Giovanella), New York: Academic Press, p. 187.

SHIMOSATO, Y., KAMEYA, T., NAGAI, K. & 4 others. (1976). Transplantation of human tumours in nude mice. *J. Natl Cancer Inst.*, **56**, 1251.

SHORTHOUSE, A.J., PECKHAM, M.J., SMYTH, J.F. & STEEL, G.G. (1980). The therapeutic response of bronchial carcinoma xenografts: a direct patient-xenograft comparison. *Br. J. Cancer*, **41**, (Suppl.) IV, 142.

ŠONKA, J., STOEHR, M., VOGT-SCHADEN, M. & VOLM, M. (1983). Isopycnic density-gradient centrifugation: a separation parameter which improves flow cytometric measurements of heterogeneous tumours. *Cytometry*, **4**, 141.

STEEL, G.G., COURTENAY, V.D. & PECKHAM, M.J. (1983). The response of chemotherapy of a variety of human tumour xenografts. *Br. J. Cancer*, **47**, 1.

STRAUS, M.J., MORAN, R.E. & SHACKNEY, S.E. (1983). Growth characteristics of lung cancer. In: *Lung Cancer. Clinical Diagnosis and Treatment*. (Ed. Straus), New York: Grune & Stratton, p. 21.

WORLD HEALTH ORGANIZATION (1981). Histological typing of lung tumours. *Tumori*, **67**, 253.